# Supplementary material for: STIM-Orai1 signaling regulates fluidity of cytoplasm during membrane blebbing
Source: Nat Commun. 2021 Jan 20;12:480. doi: 10.1038/s41467-020-20826-5 (PMC7817837; doi:10.1038/s41467-020-20826-5)
Supplement: Supplementary file 1 — Supplementary Information [file 41467_2020_20826_MOESM1_ESM.pdf]

Supplementary Information

STIM-Orai1 signaling regulates fluidity of cytoplasm during membrane blebbing

Kana Aoki [1], Shota Harada [2], Keita Kawaji [2], Kenji Matsuzawa [1], Seiichi Uchida [2] and Junichi Ikenouchi [1] *

1Department of Biology, Faculty of Sciences, Kyushu University, Fukuoka 819-0395, Japan.

2Department of Advanced Information Technology, Kyushu University, Fukuoka 819-0395, Japan.

* Address correspondence to: Junichi Ikenouchi M.D., Ph.D.

774 Motooka, Nishi-ku, Fukuoka 819-0395, Japan

E-mail: ikenouchi.junichi.033@m.kyushu-u.ac.jp

Supplementary Figure 1

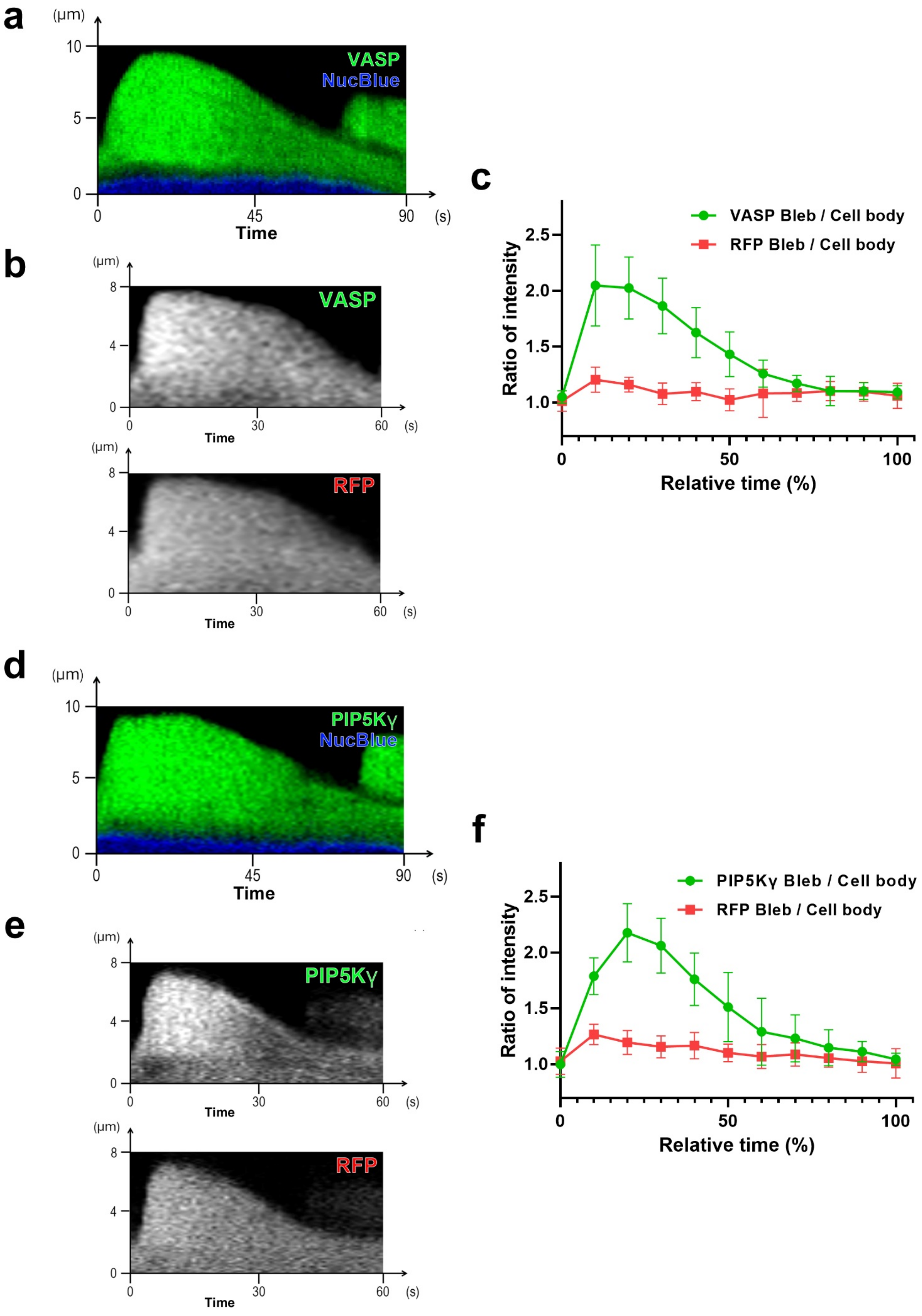

Figure S1

The protein composition of cytoplasm in expanding blebs is different from that of other cytoplasmic regions. Related to Figure 1.

a-**f** Kymograph analyses of GFP-VASP (**a**-**c**) and GFP-PIP5Kγ (**d**-**f**) in bleb cytoplasm. **a** and **d** Representative merged kymographs of NucBlue (blue) and either GFP-VASP (**a**) or GFP- PIP5Kγ (**d**) from three independent experiments. **b** and **e** Representative kymographs of the control cytoplasm protein, RFP (lower panels) and either GFP-VASP or GFP- PIP5Kγ (upper panels). **c** and **f** Fluorescence intensities of GFP-Mena and RFP were quantified following the schematic shown in **Fig. 1i**. Data presented are means ± SD based on the values from five independent experiments. **a**, **b**, **d** and **e** Bleb extension is shown on the vertical axis, and time is shown on the horizontal axis. Source data are provided as a Source Data file.

Supplementary Figure 2

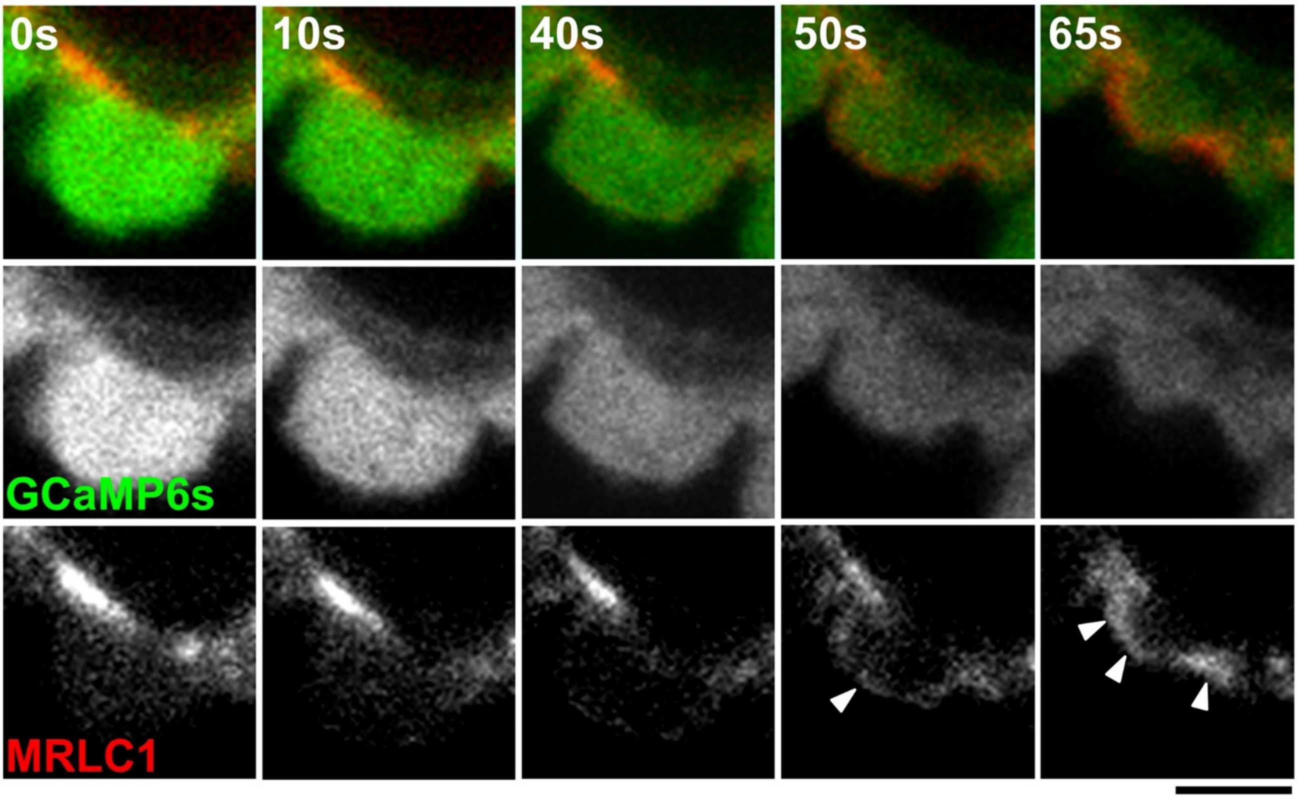

Figure S2

Myosin is recruited to the plasma membrane of the bleb after the cytoplasmic concentration of calcium ions is decreased. Related to Figure 2.

Membrane blebbing of DLD1 cells expressing GCaMP6s and Scarlet-myosin regulatory light chain 1 (MRLC1) from five independent experiments. Arrowheads show MRLC1 recruitment to the plasma membrane. Scale bar, 2 μm.

Supplementary Figure 3

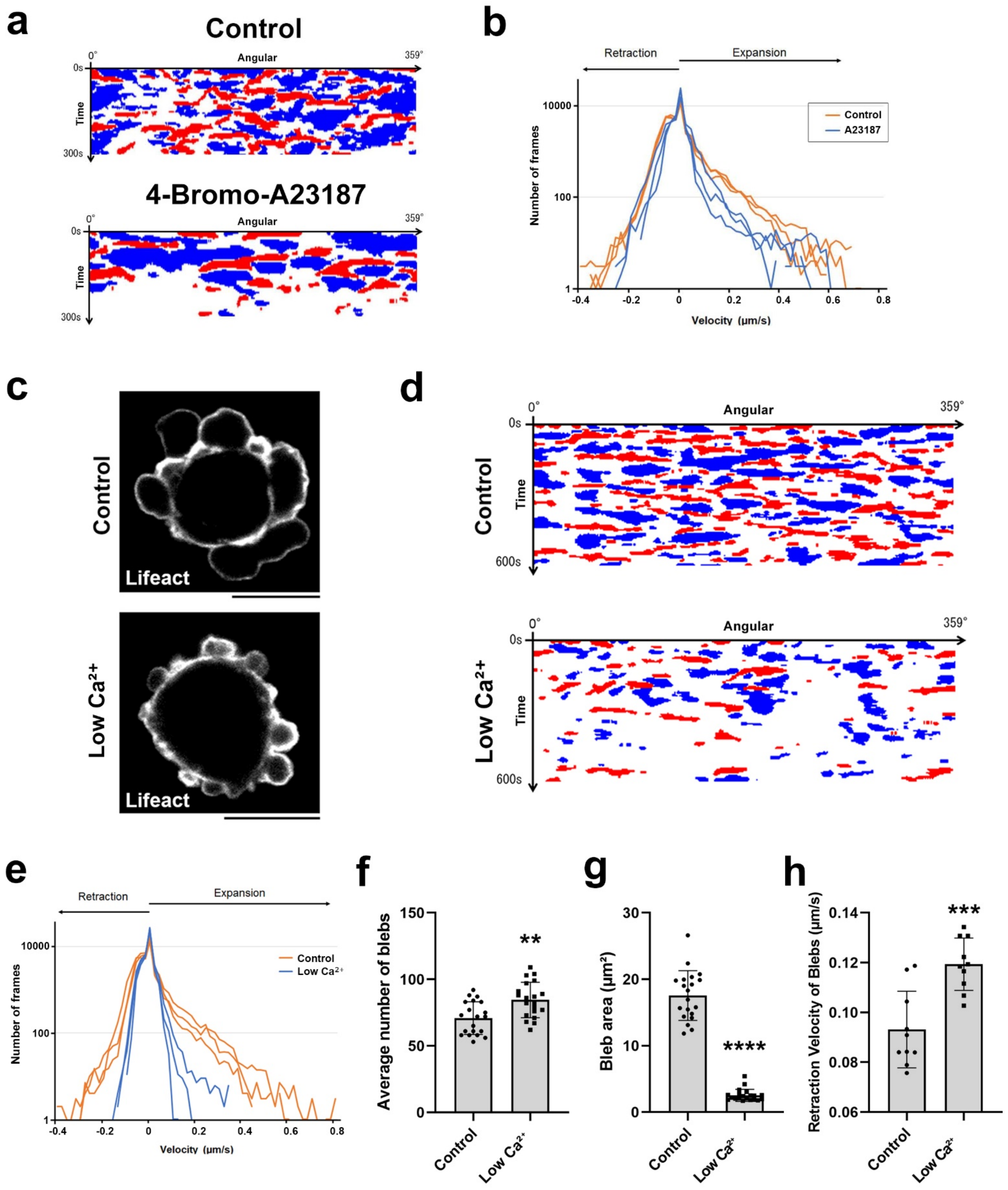

Figure S3

Reduction of extracellular calcium ion concentration suppresses the expansion of blebs. Related to Figure 3.

a Tricolor maps showing angular coordinates along the horizontal axis and time on the vertical axis. Red zones represent expansion, blue zones represent retraction, and white zones represent no movement. Vehicle-treated control cells are shown in the upper panel and 4-bromo-A23187-treated (10 μM) cells are shown in the lower panel. Results shown are representative of three independent experiments per condition. **b** Histograms of bleb expansion and retraction velocities in cells shown in **a**. Three independent measurements are plotted for each condition. **c** Representative membrane blebbing in DLD1 cells expressing Lifeact-RFP cultured in control medium (upper panel) or low calcium medium (Low Ca^{2+}; lower panel) from three independent experiments. **d** Tricolor maps of the cells shown in **c**. **e** Histograms of bleb expansion and retraction velocities in cells shown in **c**. The number (**f**, N=20 cells), area (**g**, N=20 blebs) and retraction velocity (**h**, N=20 blebs) of membrane blebs in cells shown in **c**. Individual data points are plotted with the means ± SD. $**P < 0.01$ (**f**), $****P < 0.0001$ (**g**) or **h** $***P < 0.001$ (Two-sided, unpaired student's t test). Source data are provided as a Source Data file.

Supplementary Figure 4

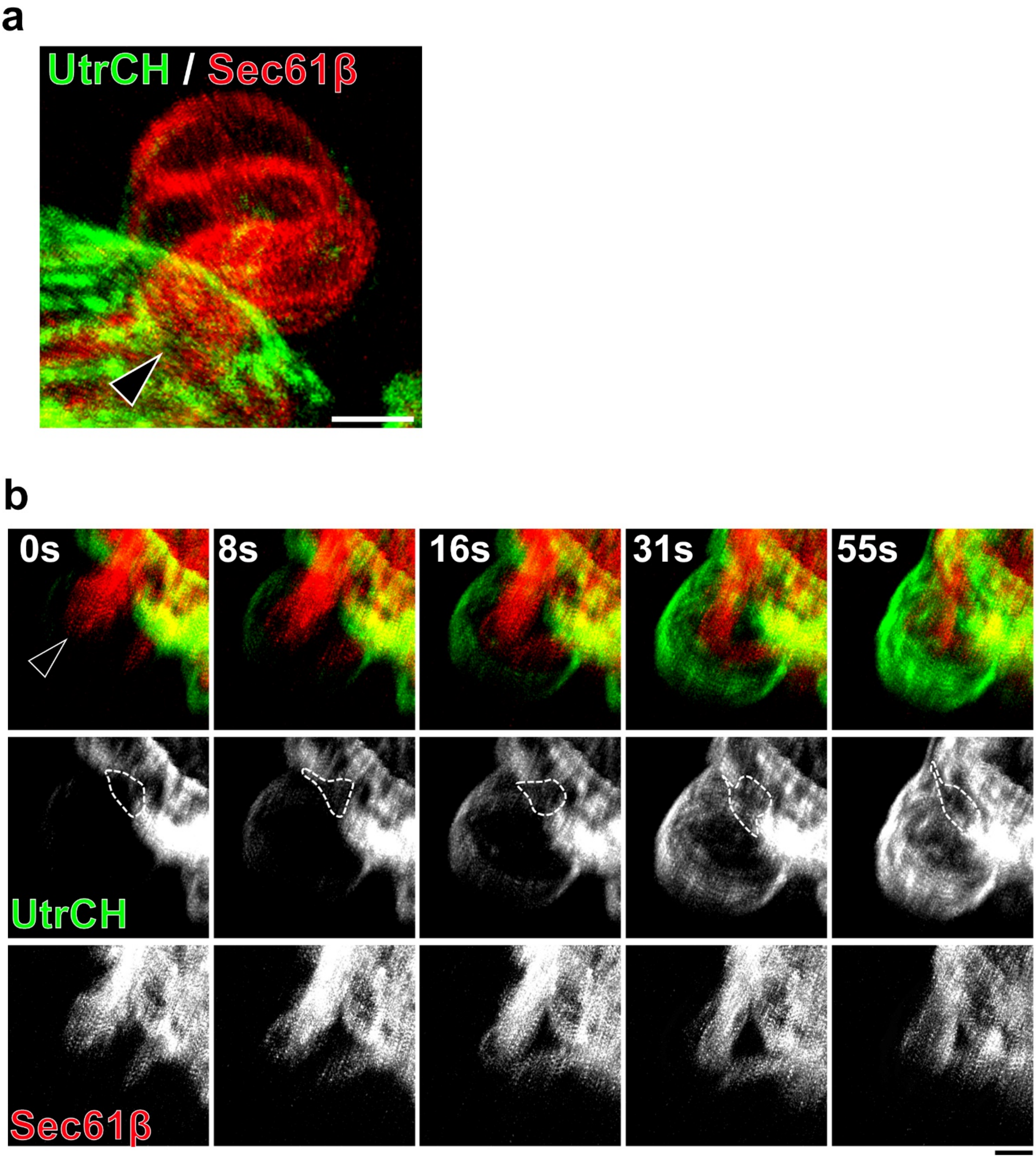

Figure S4

ER flow into blebs through the gap of actin cortex during bleb expansion. Related to Figure 4.

a Representative still image of membrane blebbing in DLD1 cells expressing Sec61β-mCherry and GFP-tagged Calponin homology domain of utrophin (UtrCH). Image shown is a 3D reconstruction of image stacks obtained using a spinning disk confocal microscope. Arrowhead shows the ER flowing into the bleb through the gap in the actin cortex. **b** Representative time lapse images of membrane blebbing in DLD1 cells expressing Sec61β-mCherry and GFP-UtrCH imaged by 3D reconstructions of spinning-disk microscopy stacks. Times shown are relative to the first image. Arrowhead shows the ER flowing into the bleb and the area enclosed by the white broken line shows the gap in the actin cortex. Results shown are representative of three independent experiments. Scale bar: 2 μm.

Supplementary Figure 5

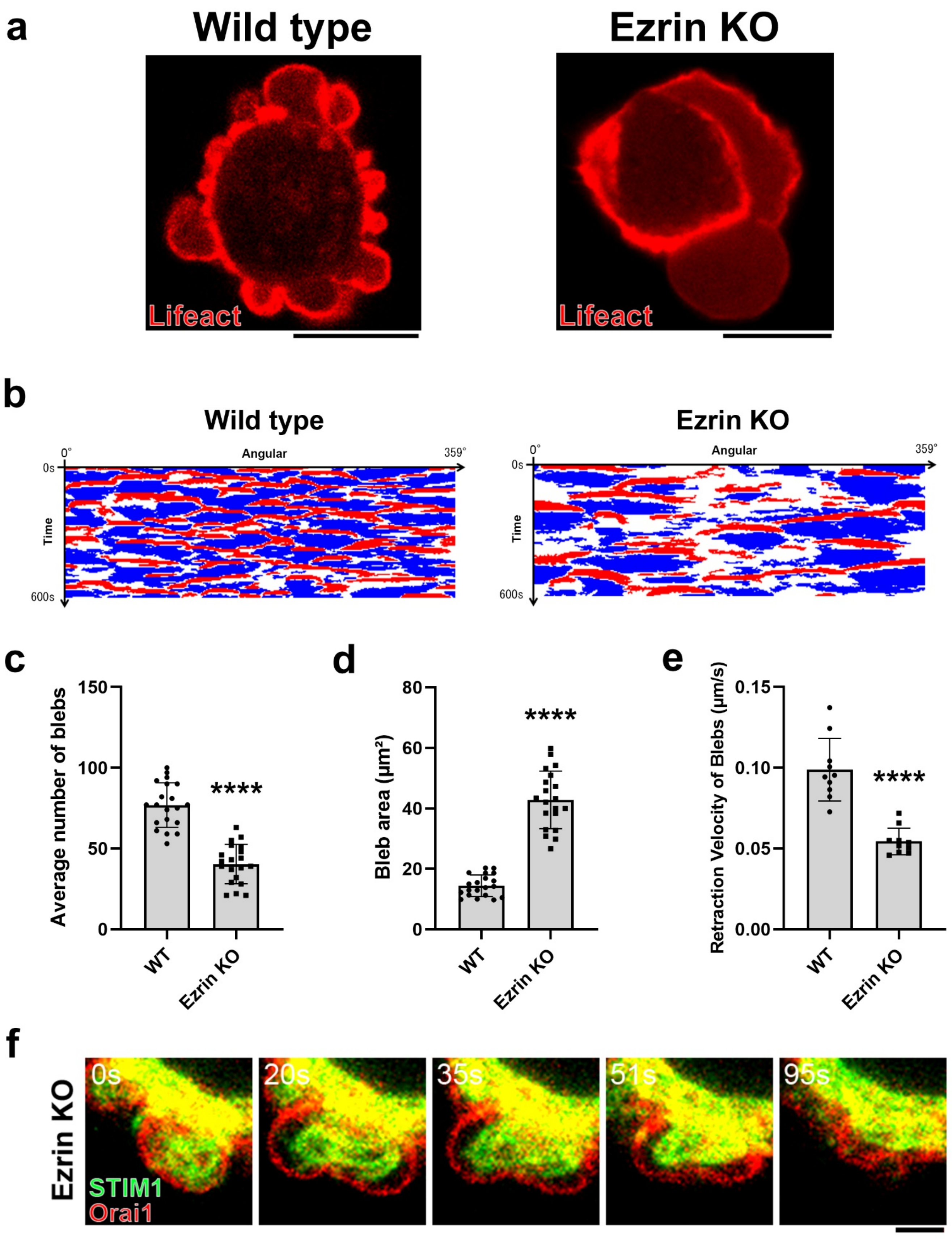

Figure S5

Ezrin knock out (KO) cells form enlarged blebs. Related to Figure 6.

a Representative still images of membrane blebbing in WT (left panel) and Ezrin KO (right panel) cells expressing Lifeact-RFP. Results shown are representative of three independent experiments. Scale bar, 10 μm. **b** Tricolor maps of cells shown in **a**. Angular coordinates are plotted along the horizontal axis and time on the vertical axis. Red zones represent expansion, blue zones represent retraction, and white zones represent no movement. The number (**c**, N=20 cells), area (**d**, N=20 blebs) and retraction velocity (**e**, N=20 blebs) of membrane blebs in cells shown in **a**. Individual data points are plotted with the means ± SD. ****P < 0.0001 (Two-sided, unpaired student's t test). Source data are provided as a Source Data file. **f** Representative membrane blebbing in ezrin KO cells expressing Orai1-mCherry and GFP-STIM1 from three independent experiments. Indicated times are relative to the first image. Scale bar, 2 μm.

Supplementary Figure 6

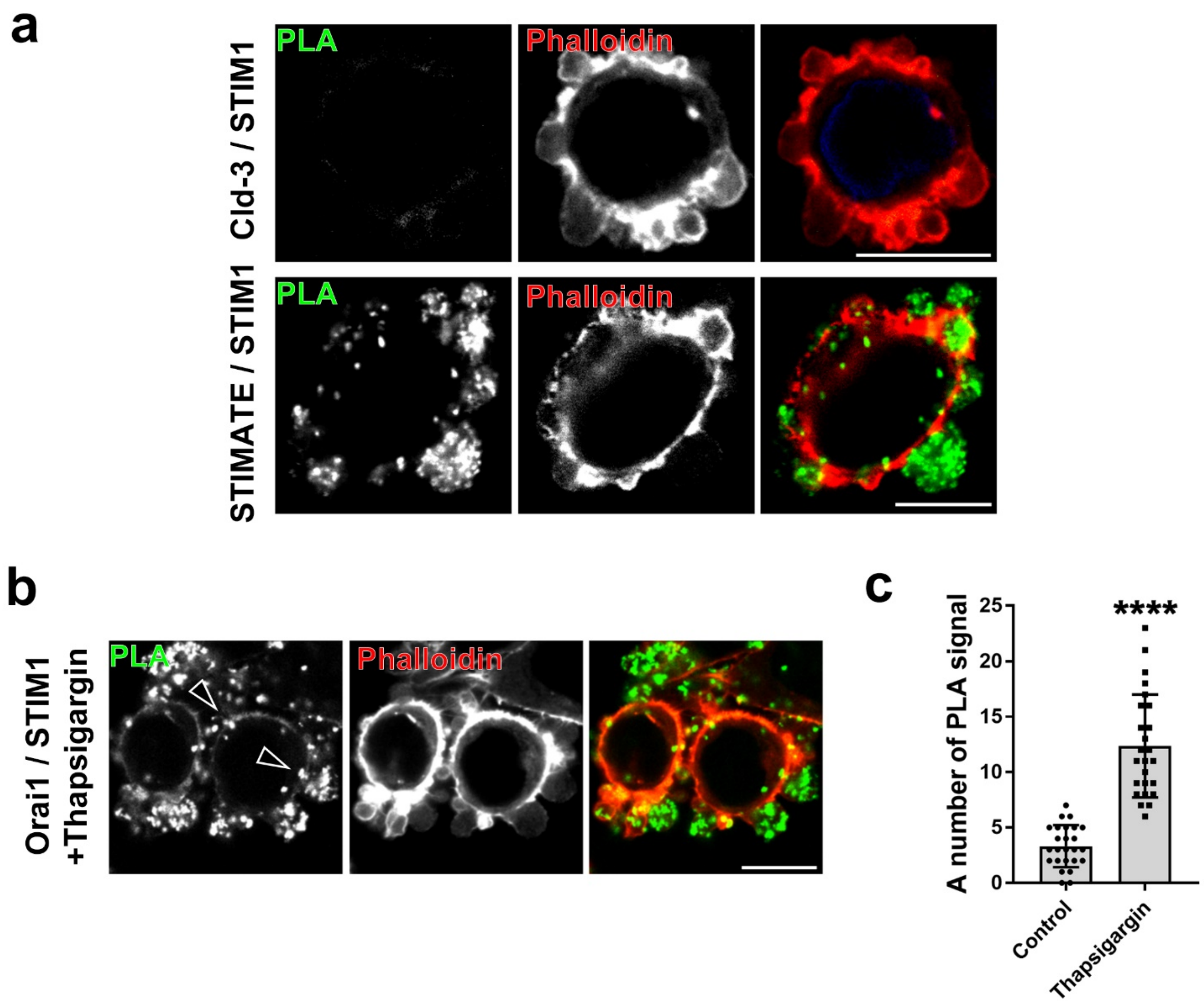

Figure S6

Proximity ligation assay (PLA) between STIM1 and Orai1. Related to Figure 6.

a Control experiments for the PLA quantified in **Figure 6h**. Upper panels show the negative control pair, Cld-3-STIM1 and lower panels show the positive control pair (STIMATE-STIM1). PLA signals (green) and actin filaments visualized by phalloidin staining (red) and merge images are shown. Images shown are representative of five independent experiments. **b** and **c** PLA for the interaction between Orai1 and STIM1 in DLD1 cells treated with Thapsigargin (1μM) for 5 min. Representative images (Scale bar, 10 μm) from five independent experiments are shown in **b** and quantifications of PLA signals in 25 independent cells are shown in **c.** Arrowheads in **b** show PLA signals outside expanding blebs. Individual data points are plotted with the means ± SD in **c**. ****P < 0.0001 (Two-sided, unpaired student's t test). Source data are provided as a Source Data file.

Supplementary Figure 7

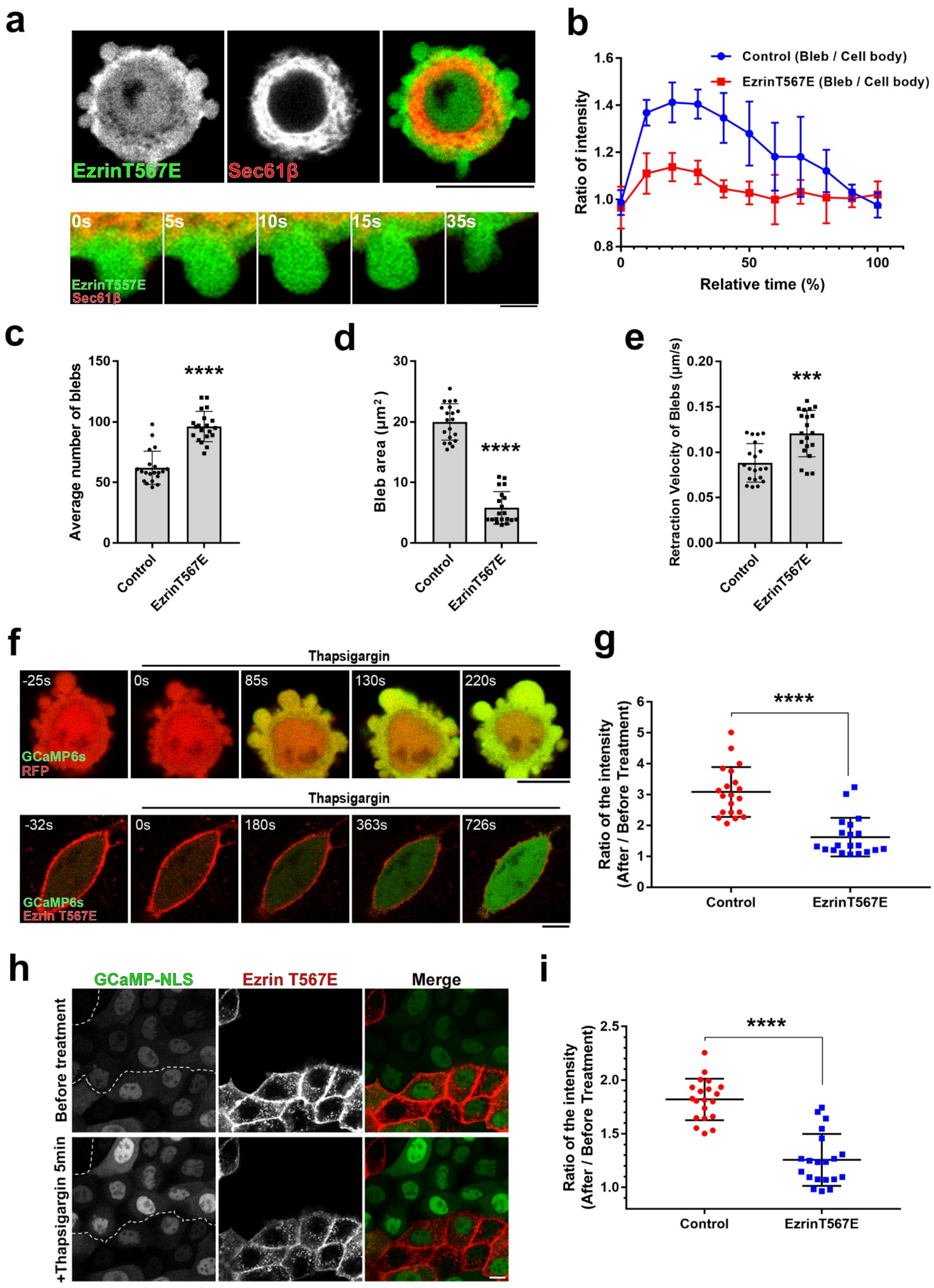

Figure S7

Ezrin T567E suppresses the formation of ER-PM contact sites. Related to Figure 6.

a-**e** Membrane blebbing in DLD1 cells expressing GFP-Ezrin T567E and mcherry-Sec61β. Blebbing was induced by treatment with the actin polymerization inhibitor Cytochalasin D (5μM) for 10 min. **a** Representative images from three independent experiments. Times shown are relative to the first image. Scale bar, 10 μm (upper panels) or 2 μm (lower panels). **b** Fluorescence intensities of GFP-Mena and RFP were quantified following the schematic shown in **Fig. 1i**. Data presented are means ± SD based on the values from five independent experiments. The number (**c**, N=20 cells), area (**d**, N=20 blebs) and retraction velocity (**e**, N=20 blebs) of membrane blebs in cells presented in **a**. Individual data points are plotted with the means ± SD. **f** Representative membrane blebbing in cells expressing GCaMP6s with either RFP (control) or Scarlet-Ezrin T567E (Ezrin T567E) and treated with Thapsigargin. Indicated times are relative to drug treatment. **g** Fluorescence intensity of GCaMP6s in were measured in the same cell both before and after Thapsigargin treatment and their ratio were plotted. Individual data points from 20 independently measured cells are shown with the means ± SD. **h** Representative images of a co-culture of MDCKII cells expressing GCaMP6s fused to a nuclear localization signal (GCaMP-NLS) alone or with Scarlet-Ezrin T567E. Upper and lower panels show cells before (upper panels) and after (lower panels) Thapsigargin treatment. Result shown is representative of five independent experiments. Scale bar, 10 μm. **i** Fluorescence intensity of GCaMP6s in were measured in the same cell both before and after Thapsigargin treatment and their ratio were plotted. Individual data points from 20 independently measured cells are shown with the means ± SD. **c**, **d**, **g**, **i** ****P < 0.0001; **e** ***P < 0.001 (Two-sided, unpaired student's t test).

Supplemental Table 1

Human STIM1 Forward	ttGAATTCGCCACCatggatgtatgcgtccgtcttgc
Human STIM1 Reverse	tcGAATTCcttcttaagaggcttcttaaag
Human Orai1 Forward	gaGAATTCGCCACCatgcatccggagcccgccccgccc
Human Orai1 Reverse	atGAATTCggcatagtggctgccgggcgtcagg
Human Mena Forward	cgGAATTCatgagtgaacagagtatctgtcag
Human Mena Reverse	acGAATTCctatgcagtatttgacttgctcag
Human VASP Forward	cgGAATTCatgagcgagacggtcatctgttcc
Human VASP Reverse	acGAATTCtcagggagaaccccgcttcctcag
Human MRLC1 Forward	gcGAATTCatgtccagcaagcgggccaaagcc
Human MRLC1 Reverse	acGAATTCctagtcgtctttatccttggcgcc
Human Ezrin Forward	gcGAATTCatgccgaaaccaatcaatgtcc
Human Ezrin Reverse	gcGAATTCttacagggcctcgaactcgtcg
Human STIMATE Forward	gcGAATTCGCCACCatgcagggccccgccgggaacg
Human STIMATE Reverse	gcGAATTCtacgggtagcccaaagcggtgc
Human Orai1E106Q mutagenesis Forward	catggtggcaatggtgCAggtgcagctggacgctg
Human Orai1E106Q mutagenesis Reverse	cagcgtccagctgcaccTGcaccattgccaccatg
Human STIM1 D76A mutagenesis Forward	ccacaaactgatggCcgatgatgccaatgg
Human STIM1 D76A mutagenesis Reverse	ccattggcatcatcgGccatcagtttgtgg
Human Ezrin T567E mutagenesis Forward	cgggacaagtacaagGAgctgcggcagatccg
Human Ezrin T567E mutagenesis Reverse	cggatctgccgcagcTCcttgtacttgtcccg
