# Supplementary material for: STIM-Orai1 signaling regulates fluidity of cytoplasm during membrane blebbing
Source: Nat Commun. 2021 Jan 20;12:480. doi: 10.1038/s41467-020-20826-5 (PMC7817837; doi:10.1038/s41467-020-20826-5)
Supplement: Supplementary file 3 — Description of Additional Supplementary Files [file 41467_2020_20826_MOESM3_ESM.pdf]

Title: Supplementary Movie 1.

Description: Trajectories of QDs during membrane blebbing.

Trajectories of QDs within the cytoplasms of expanding and retracting blebs. DLD1 cells were transfected with RFP-tagged Lifeact. Color coding of the trajectories follow Fig. 1C. QDs trajectories were acquired according to the method described in “Tracking of particle trajectories” section. Frames were taken every 200 ms. (Scale bar, 2 μm.)

Title: Supplementary Movie 2.

Description: Mena is enriched in the cytoplasm of expanding blebs.

DLD1 cells expressing GFP-tagged Mena were imaged. Frames were taken every 2s. (Scale bar, 2 μm.)

Title: Supplementary Movie 3.

Description: The concentration of calcium ions in the cytoplasm of the expanding bleb is higher than that of other cytoplasmic region.

DLD1 cells expressing GCaMP6s and live stained with NucBlue (nucleus) were imaged. Frames were taken every 2s. (Scale bar, 2 μm.)

Title: Supplementary Movie 4.

Description: The bleb expansion period was prolonged by the increasing of the influx of calcium ion.

Membrane blebbing of DLD1 cells transfected with Lifeact–RFP and GCaMP6s. Cells were treated with 4-bromo-A23187 at t = 0. (Scale bar, 10 μm.)

Title: Supplementary Movie 5.

Description: The ER-PM contact sites are formed only during the expanding blebs.

DLD1 cells expressing GFP-tagged PLCδ-PH and Sec61β-mCherry were imaged. The arrowheads show the ER-PM contact sites. Frames were taken every 5s. (Scale bar, 2 μm.)

Title: Supplementary Movie 6.

Description: Formation of ER-PM contact sites are inhibited by the accumulation of actin cytoskeleton in retracting blebs.

DLD1 cells expressing GFP-tagged E-Syt1 and Lifeact–RFP were imaged. The arrowheads show the ER-PM contact sites. Frames were taken every 2s. (Scale bar, 2 μm.)

Title: Supplementary Movie 7.

Description: ER-PM contact sites are increased by the inhibition of actin polymerization

beneath the plasma membrane.

DLD1 cells expressing GFP-tagged E-Syt1 and Lifeact–RFP were imaged. Cells were treated with 1μM Latrunculin B at t = 0. ER-PM contact sites persist at the actin cytoskeleton-free protruded membranes (arrowheads). (Scale bar, 10 μm.).
