# Supplementary material for: STIM-Orai1 signaling regulates fluidity of cytoplasm during membrane blebbing
Source: Nat Commun. 2021 Jan 20;12:480. doi: 10.1038/s41467-020-20826-5 (PMC7817837; doi:10.1038/s41467-020-20826-5)
Supplement: Supplementary file 11 — Reporting Summary [file 41467_2020_20826_MOESM11_ESM.pdf]

nature research

Corresponding author(s): Junichi Ikenouchi

Last updated by author(s): Dec 8, 2020

Reporting Summary

Nature Research wishes to improve the reproducibility of the work that we publish. This form provides structure for consistency and transparency in reporting. For further information on Nature Research policies, see our Editorial Policies and the Editorial Policy Checklist.

Statistics

For all statistical analyses, confirm that the following items are present in the figure legend, table legend, main text, or Methods section.

n/a	Confirmed	
☐	☒	The exact sample size (n) for each experimental group/condition, given as a discrete number and unit of measurement
☐	☒	A statement on whether measurements were taken from distinct samples or whether the same sample was measured repeatedly
☐	☒	The statistical test(s) used AND whether they are one- or two-sided<br>*Only common tests should be described solely by name; describe more complex techniques in the Methods section.*
☒	☐	A description of all covariates tested
☐	☒	A description of any assumptions or corrections, such as tests of normality and adjustment for multiple comparisons
☐	☒	A full description of the statistical parameters including central tendency (e.g. means) or other basic estimates (e.g. regression coefficient) AND variation (e.g. standard deviation) or associated estimates of uncertainty (e.g. confidence intervals)
☐	☒	For null hypothesis testing, the test statistic (e.g. F, t, r) with confidence intervals, effect sizes, degrees of freedom and P value noted<br>*Give P values as exact values whenever suitable.*
☒	☐	For Bayesian analysis, information on the choice of priors and Markov chain Monte Carlo settings
☒	☐	For hierarchical and complex designs, identification of the appropriate level for tests and full reporting of outcomes
☒	☐	Estimates of effect sizes (e.g. Cohen's d, Pearson's r), indicating how they were calculated

Our web collection on statistics for biologists contains articles on many of the points above.

Software and code

Policy information about availability of computer code

Data collection	ZEN2012 (Zeiss) was used to collect data from confocal microscopy. Fusion software (OXFORD Instruments) was used to capture and acquire data from spinning-disk confocal microscopy (Dragonfly200; OXFORD Instruments) .
Data analysis	Cell image analysis was performed with ImageJ. Visualization and quantitative analysis of membrane bleb dynamics was performed as previously described (Aoki et al. PNAS 2016). Tracking analysis of particle trajectories was performed by applying the stable marriage algorithm. The code used in this study are available from Github (https://github.com/uchidalab/bleb-analysis).<br>GraphPad Prism 8.4.1 (GraphPad Software) was used to graph data and to perform statistical analyses.

For manuscripts utilizing custom algorithms or software that are central to the research but not yet described in published literature, software must be made available to editors and reviewers. We strongly encourage code deposition in a community repository (e.g. GitHub). See the Nature Research guidelines for submitting code & software for further information.

Data

Policy information about availability of data

All manuscripts must include a data availability statement. This statement should provide the following information, where applicable:

- Accession codes, unique identifiers, or web links for publicly available datasets
- A list of figures that have associated raw data
- A description of any restrictions on data availability

Raw data associated with figures are included in the Source Data file.

Field-specific reporting

Please select the one below that is the best fit for your research. If you are not sure, read the appropriate sections before making your selection.

- [x] Life sciences
- [ ] Behavioural & social sciences
- [ ] Ecological, evolutionary & environmental sciences

For a reference copy of the document with all sections, see nature.com/documents/nr-reporting-summary-flat.pdf

Life sciences study design

All studies must disclose on these points even when the disclosure is negative.

Sample size	The sample size was chosen in advance on the basis of common practice of the described experiment and is mentioned for each experiment. No statistical methods were used to predetermine sample size. We ensured that sample sizes were sufficient by checking that inclusion of additional data points did not significantly change the variance (SD) of the data.
Data exclusions	No data were excluded.
Replication	All experimental results were replicated at least 3 times with enough reproducibility. Therefore, attempts of data replication were successful.
Randomization	Animals and humans were not used in the study. Randomization is not relevant as the same cell lines were used in all cases.
Blinding	Blinding is not applicable to data collection. In the case of time-lapse imaging analysis, all labels were removed and individual microscopy files were analyzed blindly in ImageJ.

Reporting for specific materials, systems and methods

We require information from authors about some types of materials, experimental systems and methods used in many studies. Here, indicate whether each material, system or method listed is relevant to your study. If you are not sure if a list item applies to your research, read the appropriate section before selecting a response.

Materials & experimental systems

n/a	Involved in the study	
☐	☒	Antibodies
☐	☒	Eukaryotic cell lines
☒	☐	Palaeontology and archaeology
☒	☐	Animals and other organisms
☒	☐	Human research participants
☒	☐	Clinical data
☒	☐	Dual use research of concern

Methods

n/a	Involved in the study	
☒	☐	ChIP-seq
☒	☐	Flow cytometry
☒	☐	MRI-based neuroimaging

Antibodies

Antibodies used	Primary antibodies were as follows: Mouse anti-GFP antibody (#11814460001, Roche); Mouse anti DYKDDDDK tag, monoclonal antibody (#014-22383, FUJIFILM Wako Pure Chemical, Osaka, Japan); Rabbit anti-HA-tag antibody (#561, MBL, Nagoya, Japan); Rabbit anti Claudin-3 polyclonal antibody (#34-1700, Invitrogen).<br>Secondary antibodies were as follows: Goat Anti-Rabbit IgG-HRP (#4030-05, Southern Biotech); Goat anti-Mouse IgG-heavy and light chain cross-adsorbed Antibody HRP Conjugate (#A90-516P, Bethyl Laboratories); Alexa Fluor 555 Phalloidin (#A34055, Thermo Fisher Scientific).
Validation	Only commercially available antibodies were used. They were all validated by the manufacturers.

Eukaryotic cell lines

Policy information about cell lines

Cell line source(s)	DLD1 cells, MDCK II cells,HEK293 cells and 4T1 cells were purchased from ATCC.
Authentication	No
Mycoplasma contamination	No
Commonly misidentified lines (See ICLAC register)	No commonly misidentified cell lines were used.
