# Supplementary material for: STIM-Orai1 signaling regulates fluidity of cytoplasm during membrane blebbing
Source: Nat Commun. 2021 Jan 20;12:480. doi: 10.1038/s41467-020-20826-5 (PMC7817837; doi:10.1038/s41467-020-20826-5)
Supplement: Supplementary file 12 — Source Data [file 41467_2020_20826_MOESM12_ESM.zip › Source File Explanation.docx]

The excel file ‘Source Data’ contains the raw data pertaining to the relevant figures in separate tabs. The data contained within each tab are briefly described below.

**Figure1b**

The numbers of events for the corresponding bleb expansion/retraction velocities (column headers) are tabulated.

**Figure1c**

The components of data obtained from tracking Quantum dot (QD) particle motion over 30 successive frames are shown as follows:

x_cell_[1^st^#]_[2^nd^#]：X-coordinate. In the following column headers, the 1^st^ number is cell number and the second number designates the particle within that cell.

y_cell_[1^st^#]_[2^nd^#]：Y-coordinate.

color_cell_[1^st^#]_[2^nd^#]：Color designation based on average velocity (Red，Yellow，Blue).

**Figure1d**

The total distance of particle motion during bleb expansion and retraction were calculated from the tracking data in Figure1c and tabulated.

**Figure1e**

MSD of 5 representative particle tracks from Figure1c are tabulated.

**Figure1f**

Diffusion coefficients were calculated from the fitted linear regression lines for each tracked particle from Figure1c. The data for bleb expansion and retraction are separately tabulated.

**Figure1k**

The ratios of bleb-to-cell body fluorescence intensities of GFP-Mena and RFP are tabulated. Each row represents the percent time relative to the duration of the time lapse.

**Figure2d**

The ratios of bleb-to-cell body fluorescence intensities of GCaMP6s and RFP are tabulated. Each row represents the percent time relative to the duration of the time lapse.

**Figure2f**

The ratios of plasma membrane fluorescence intensities of GCaMP6s-CAAX and mCherry-PLCδ-PH are tabulated. Each row represents the percent time relative to the duration of the time lapse.

**Figure3b**

The components of data obtained from tracking QD particle motion over 30 successive frames are tabulated for control and 4-bromo-A23187-treated cells. As in Figure1c, the 1^st^ number is cell number and the second number designates the particle within that cell.

x_cell_[1^st^#]_[2^nd^#]：X-coordinate. In the following column headers, the 1^st^ number is cell number and the second number designates the particle within that cell.

y_cell_[1^st^#]_[2^nd^#]：Y-coordinate.

color_cell_[1^st^#]_[2^nd^#]：Color designation based on average velocity (Red，Yellow，Blue)

**Figure3c**

The total distance of particle motion during bleb expansion and retraction were calculated from the tracking data in Figure3b and tabulated.

**Figure3d**

MSD of 5 representative particle tracks from Figure3b are tabulated.

**Figure3e**

Diffusion coefficients were calculated from the fitted linear regression lines for each tracked particle in Figure3b. The data for control and 4-Bromo-A23187-treated cells are separately tabulated.

**Figure3f**

The numbers of blebs formed over a 10-minute period in control and 4-Bromo-A23187-treated cells are tabulated.

**Figure3g**

The areas of maximally expanded blebs for control and 4-Bromo-A23187-treated cells are tabulated.

**Figure3h**

Bleb retraction velocities were calculated from the time of maximum bleb expansion to retraction termination. The data for control and 4-Bromo-A23187-treated cells are separately tabulated.

**Figure4d**

The numbers of blebs formed over a 10-minute period in control, AnCoA4- and SKF96365-treated cells are tabulated.

**Figure4e**

The areas of maximally expanded blebs for control, AnCoA4- and SKF9636-treated cells are tabulated.

**Figure4f**

Bleb retraction velocities for control, AnCoA4- and SKF9636-treated cells are tabulated. Bleb retraction velocities were calculated as in Figure3h.

**Figure5c**

The ratios of bleb-to-cell body fluorescence intensities of GCaMP6s and RFP are tabulated for DLD1 Orai1 WT and Orai1 E106Q over-expressing cells.

**Figure5d**

The numbers of blebs formed over a 10-minute period in DLD1 Orai1 WT and Orai1 E106Q over-expressing cells are tabulated.

**Figure5e**

The areas of maximally expanded blebs for DLD1 Orai1 WT and Orai1 E106Q over-expressing cells are tabulated.

**Figure5f**

Bleb retraction velocities for DLD1 Orai1 WT and Orai1 E106Q over-expressing cells were calculated as in Figure3h and tabulated.

**Figure5i**

The numbers of events for the corresponding bleb expansion/retraction velocities are tabulated for each of the STIM-1 WT- and D76A-expressing cells.

**Figure5j**

The numbers of blebs formed over a 10-minute period in STIM-1 WT- and D76A-expressing cells are tabulated.

**Figure5k**

The areas of maximally expanded blebs for STIM-1 WT- and D76A-expressing cells are tabulated.

**Figure5l**

Bleb retraction velocities for STIM-1 WT- and D76A-expressing cells were calculated as in Figure3h and tabulated.

**Figure6d/6e (Full scan)**

Full membranes of the immunoblots processed for the figure are shown.

**Figure6f**

Full membranes of the immunoblots used for the figure (indicated) and quantitative analyses are shown together with the densitometry data. Arrows indicate the quantified bands where ambiguous.

**Figure6h**

The numbers of PLA signals expanding and retracting blebs are tabulated.

**Figure7a**

The numbers of migrated cells in 10 fields of view were averaged and tabulated for control, Orai1 E106Q and EzrinT567E over-expressing cells. Independent experiments are shown per row.

**Figure7d**

The numbers of cells exhibiting single cell, amoeboid migration in the experiment shown in Figure7c are annotated for control, AnCoA4- and SKF96365-treated cells are shown.

**FigureS1c**

The ratios of bleb-to-cell body fluorescence intensities of GFP-VASP and RFP are tabulated. Each row represents the percent time relative to the duration of the time lapse.

**FigureS1f**

The ratios of bleb-to-cell body fluorescence intensities of PIP5K gamma-GFP and RFP are tabulated. Each row represents the percent time relative to the duration of the time lapse.

**FigureS3b**

The numbers of events for the corresponding bleb expansion/retraction velocities are tabulated for control and 4-bromo-A23187-treated cells.

**FigureS3e**

The numbers of events for the corresponding bleb expansion/retraction velocities are tabulated for control and low-calcium-cultured cells.

**FigureS3f**

The numbers of blebs formed over a 10-minute period in DLD1 cells cultured in control and low-calcium medium are tabulated.

**FigureS3g**

The areas of maximally expanded blebs for DLD1 cells cultured in control and low-calcium medium are tabulated.

**FigureS3h**

Bleb retraction velocities for DLD1 cells cultured in control and low-calcium medium were calculated as in Figure3h and tabulated.

**FigureS5c**

The numbers of blebs formed over a 10-minute period in DLD1 WT and Ezrin KO cells are tabulated.

**FigureS5d**

The areas of maximally expanded blebs for DLD1 WT and Ezrin KO cells are tabulated.

**FigureS5e**

Bleb retraction velocities for DLD1 WT and Ezrin KO cells were calculated as in Figure3h and tabulated.

**FigureS6c**

The numbers of PLA signals in control cells and thapsigargin-treated cells are tabulated.

**FigureS7b**

The ratios of bleb-to-cell body fluorescence intensities of GCaMP6s and RFP for DLD1 WT and Ezrin T567E over-expressing cells are tabulated. Each row represents the percent time relative to the duration of the time lapse.

**FigureS7c**

The numbers of blebs formed over a 10-minute period in DLD1 WT and Ezrin T567E over-expressing cells treated with 5 µM Cytochalasin D are tabulated.

**FigureS7d**

The areas of maximally expanded blebs for DLD1 WT and Ezrin T567E over-expressing cells treated with 5 µM Cytochalasin D are tabulated.

**FigureS7e**

Bleb retraction velocities for DLD1 WT and Ezrin T567E over-expressing cells treated with 5 µM Cytochalasin D were calculated as in Figure3h and tabulated

**FigureS7g**

The ratios of GCaMP6s fluorescence intensities at 5 min before-to-5 min after thapsigargin treatment in DLD1 WT and Ezrin T567E over-expressing cells are tabulated.

**FigureS7i**

The ratios of GCaMP-NLS fluorescence intensities at 5 min before-to-5 min after thapsigargin treatment in control MDCKII and Ezrin T567E over-expressing cells are tabulated.
